# State-Level Structural Racism and Subjective Cognitive Decline: Regional Variation in U.S. Adults

**DOI:** 10.1093/geroni/igaf122.4277

**Published:** 2025-12-31

**Authors:** Kun Wang, Joonhyeog Park, Suk-Young Kang, Yanjun Dong, Victoria RIzzo, Danan Gu

**Affiliations:** University of Alabama at Birmingham, Birmingham, Alabama, United States; University of Pennsylvania, Philadelphia, Pennsylvania, United States; Binghamton University, Binghamton, New York, United States; SUNY at Albany, Albany, New York, United States; University at Albany, SUNY, Albany, New York, United States; Jiangsu China, Suzhou, Jiangsu, China (People’s Republic)

## Abstract

Structural racism—geographically patterned across U.S. regions—shapes population health, yet links to cognition remain underexplored. Using 2020–2023 BRFSS data, we evaluated associations between state-level structural racism (SLSR) and subjective cognitive decline (SCD) among non-Hispanic (NH) Black and White adults aged ≥45 (N = 180,790) and assessed regional heterogeneity. SLSR (2020), a six-indicator index of socioeconomic status, healthcare access, and residential segregation, with higher values indicating larger Black–White disparities disadvantaging Black individuals, was grouped into national quartiles (Q1–Q4) for pooled analyses. Primary analyses used survey-weighted logistic regressions; sensitivity analyses used multilevel logistic models; both adjusted for individual- and state-level covariates. In pooled analyses, associations were nonmonotonic: versus Q1, Q2 had higher odds of SCD (aOR=1.15; 95%CI 1.07–1.24) and Q4 had lower odds (aOR=0.86; 95%CI 0.79–0.94). Given marked geography (Q1: South, West; Q4: Midwest, New England, Mountain West), we conducted region-stratified analyses using within-region low/medium/high SLSR categories to avoid sparse cells. Compared with low SLSR, medium and high SLSR were associated with higher odds of SCD in the South; high SLSR with higher odds in the Midwest but lower odds in the Northeast and West. These patterns were evident among NH White adults but not NH Black adults (except the South). Results were robust in adults ≥65. Region-dependent findings—especially inverse associations among NH White adults in the Northeast and West—imply stratified advantage in higher-racism settings; largely null associations for NH Black adults may reflect SCD under-detection or distinct pathways. Longitudinal, fine-grained studies using objective cognitive outcomes are needed to clarify mechanisms and guide region-tailored policy.

